# Supplementary material for: KIPEs3: Automatic annotation of biosynthesis pathways
Source: PLoS One. 2023 Nov 16;18(11):e0294342. doi: 10.1371/journal.pone.0294342 (PMC10653506; doi:10.1371/journal.pone.0294342)
Supplement: S2 File — The alignment was generated using MAFFTv7. Characteristic regions of plant LCY-bs are indicated above the sequences: Dinucleotide binding site, Cyclase motifs (CM) I and II, and Charged region. Domains described as essential for LCY-b activity are shown as LCY-b CAD (Catalytic Activity Domain). The functional LYC-b on the basis of presence of conserved amino acid residues are highlighted in blue color. (PDF) [file pone.0294342.s002.pdf]

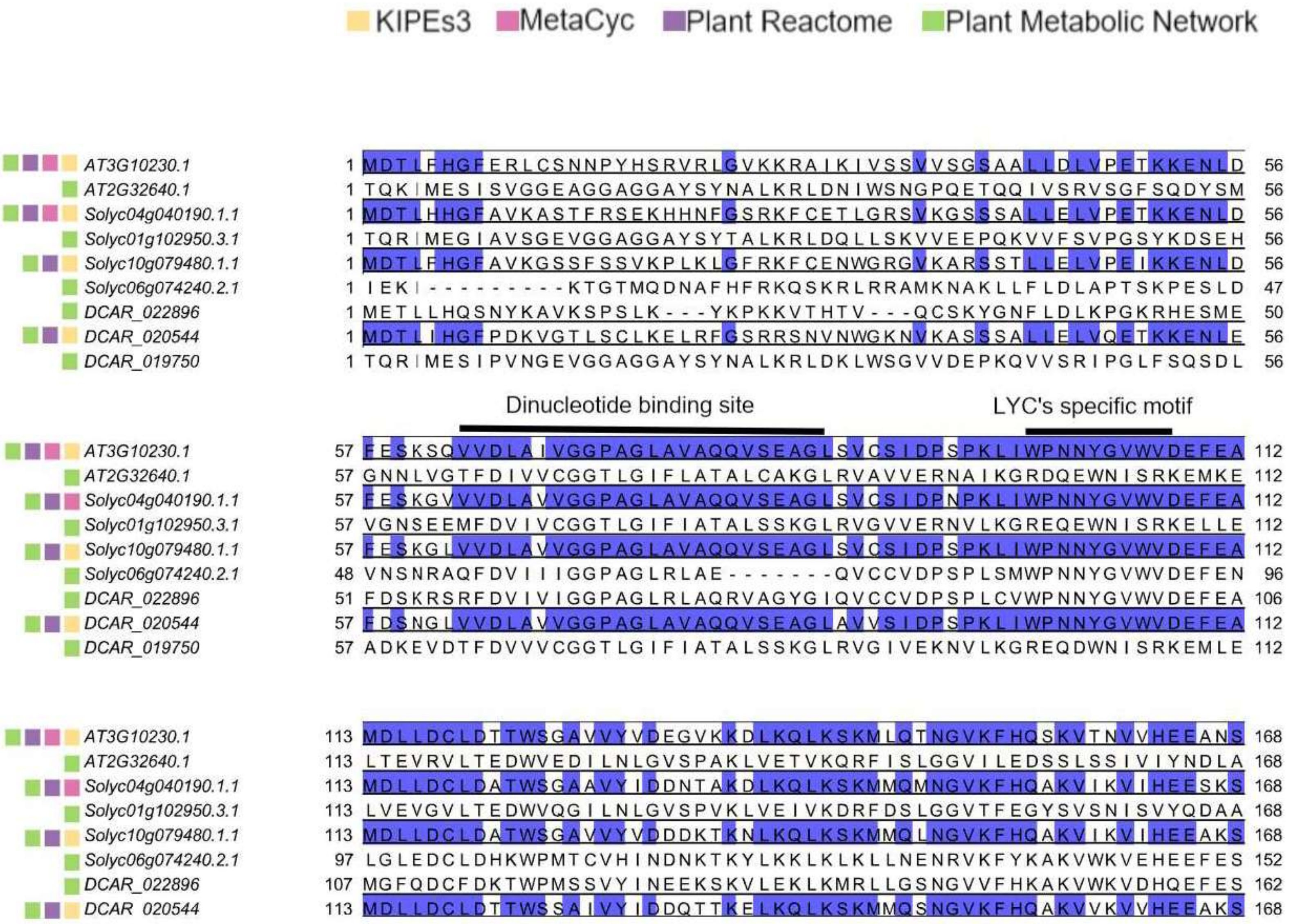
KIPEs3
MetaCyc
Plant Reactome
Plant Metabolic Network
Dinucleotide binding site
LYC's specific motif
AT3G10230.1
AT2G32640.1
Solyc04g040190.1.1
Solyc01g102950.3.1
Solyc10g079480.1.1
Solyc06g074240.2.1
DCAR_022896
DCAR_020544
DCAR_019750

DCAR_019750 113 LVEVGILEEEWVENILNLGVSPSKLIEKMKTRFNSFDGVILEGLGVSSICVYDDAA 168

AT3G10230.1 169 TVVCSDGVKIQASVVLDATGFSRCLVQYDKPYNPGYQVAYGIVAHPFDVDKMRDKH 224
AT2G32640.1 169 VMQLSKGDTLSSRLVIDAMGFSPILKQIKRGRKPGMCLVVGSCAHGFKENSSSDVI 224
Solyc04g040190.1.1 169 MLICNDGITIQATVVLDATGFSRSLVQYDKPYNPGYQVAYGILAHPFDVNKMRDSH 224
Solyc01g102950.3.1 169 VLQLKEGKTLFSRLIIDAMGFSPIVKQIRCGRKPGMCLVVGTCCRGFKENSTSDVI 224
Solyc10g079480.1.1 169 MLICSDGVTIQATVVLDATGFSRCLVQYDKPYNPGYQVAYGILAHPFDTSKMRDSH 224
Solyc06g074240.2.1 153 SIVCDDGKKIRGSLVVDASGFASDFIEYDRPRNHGYQIAHGVLVHPFDLDKMRDSH 208
DCAR_022896 163 SILCDDGKEFKASLIVDASGFASTFVDYDKPRNHGYQLAHGILAHPFELDRMRDSH 218
DCAR_020544 169 LLICNDGVTIQAAVVLDATGFSRCLVQYDKPYNPGYQVAYGIVAHPFDVNKMRDSH 224
DCAR_019750 169 ILQLDSGKRLSSRLVIDAMGFSPVVKQIRGGRKPGFCLVVGSCCRGFKDNKTSDVI 224

CMI LYC-b CAD CMI

AT3G10230.1 225 LDSYPELKERNSKIPTFLYAMPFSSNRIFLEETSLVARPGLRMEDIQERMAARLKH 280
AT2G32640.1 225 YSSSSVTRVADSNVQLFWEAFPAGSGPLFTYTEPQSTSPSLDLLEEYWKLMPKYQG 280
Solyc04g040190.1.1 225 LKNNTDLKERNSRIPTFLYAMPFSSNRIFLEETSLVARPGLRIDDIQERMVARLNH 280
Solyc01g102950.3.1 225 FSSATAKEVGQSLVQYFWEAFPAGSGPIFTYVDPQPGSPQLELLEDYWDLMPKYQG 280
Solyc10g079480.1.1 225 LNNNVKLKERNRKVPTFLYAMPFSSNRIFLEETSLVARPGLRMDDIQERMVARLSH 280
Solyc06g074240.2.1 209 LGNEPYLRVNNAKEPTFLYAMPFDRDLVFLEETSLVSRPVLSYMEVKRRMVARLRH 264
DCAR_022896 219 LGNEPALRFANAKSPTFLYAMPFDSNLIFLEETSLVSRPALSYKEVKLRMAARLRH 274
DCAR_020544 225 LNGNTELKERNSKIPTFLYAMPFSSDRIFLEETSLVARPGLAMGDIQERMVARLRH 280
DCAR_019750 225 YSSAEVMQVGESQVQYFWEAFPAGSGLMFTYVDPQPGSPKLELLEDYWNLMPDYQG 280

LYC-b CAD CMII LYC-b CAD

AT3G10230.1 281 LGINVKRIEEDERCVIPMGGPLPVLPQRVVGIGGTAGMVHPSTGYMVARTLAAAPI 336
AT2G32640.1 281 VSLDELEILRVVYGIFPTNSPLPAAFDRVLQFGDASGIQSPVSGSLTRHLGRLSNG 336
Solyc04g040190.1.1 281 LGIKVKSIEEDEHCLIPMGGPLPVLPQRVVGIGGTAGMVHPSTGYMVARTLAAAPV 336
Solyc01g102950.3.1 281 VSFDDLEILRIIFGIFPTDSPLPAAFDRILQFGDASGIQSPVSGSLTRHLGRLTTG 336
Solyc10g079480.1.1 281 LGIKVTSIEEDEQCVIPMGGPLPVIPQRVVGIGGTAGMVHPSTGYMVARTLAAAPV 336

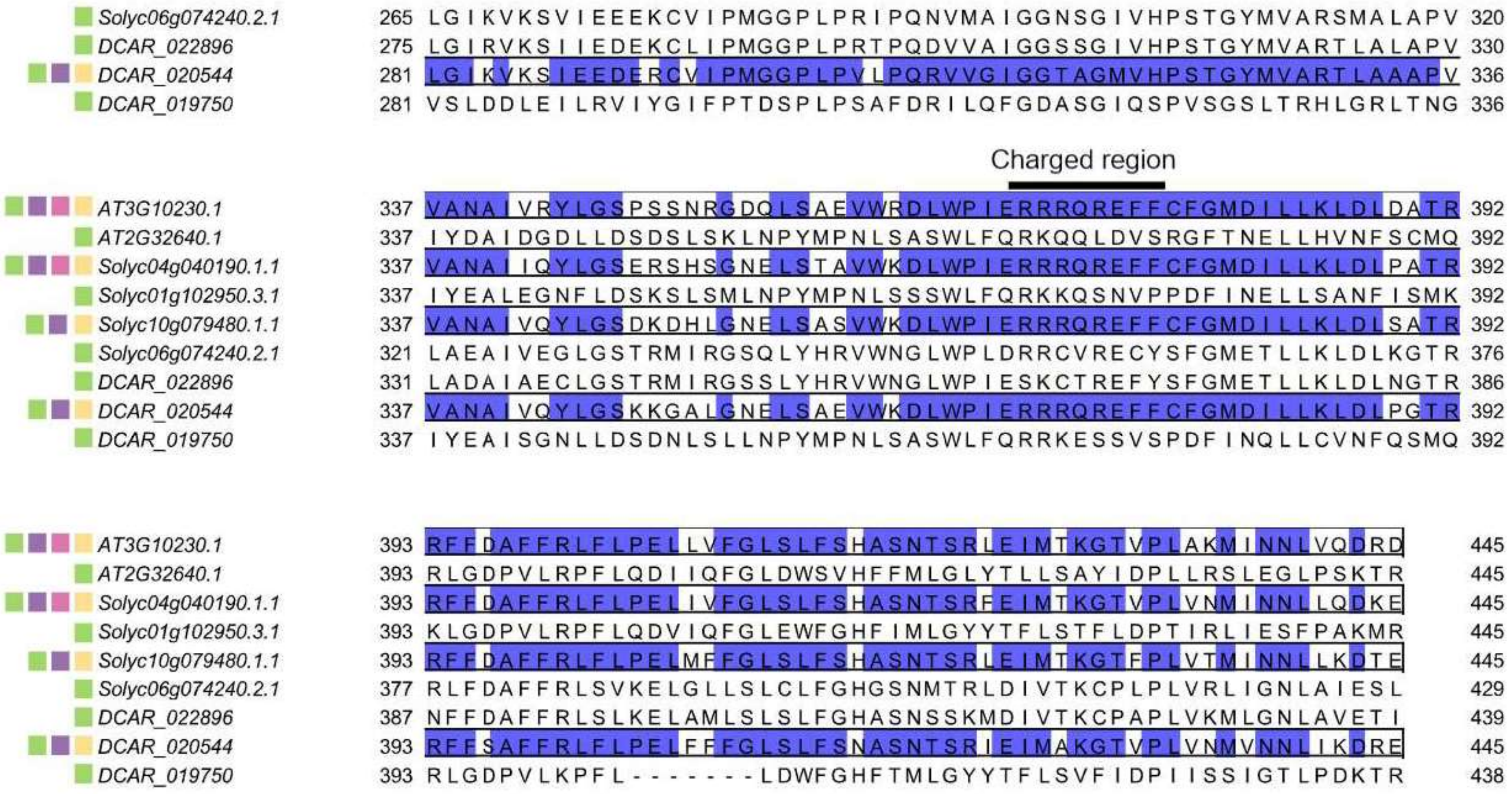
Solyc06g074240.2.1 265 LGIKVKSVIEEEKCVIPMGGPLPRIPQNVMAIGGNSGIVHPSTGYMVARSMALAPV 320
DCAR_022896 275 LGIRVKSIIEDEKCLIPMGGPLPRTPQDVVAIGGSSGIVHPSTGYMVARTLALAPV 330
DCAR_020544 281 LGIKVKSIEEDERCVIPMGGPLPVLPQRVVGIGGTAGMVHPSTGYMVARTLAAAPV 336
DCAR_019750 281 VSLDDLEILRVIYGIFPTDSPLPSAFDRILQFGDASGIQSPVSGSLTRHLGRLTNG 336
Charged region
AT3G10230.1 337 VANAIVRYLGSPSSNRGDQLSAEVWRDLWPIERRRQREFFCFGMDILLKLDLDATR 392
AT2G32640.1 337 IYDAIDGDLLDSDSLSKLNPYMPNLSASWLFQRKQQLDVSRGFTNELLHVNFSCMQ 392
Solyc04g040190.1.1 337 VANAIIQYLGSERSHSGNELSTAVWKDLWPIERRRQREFFCFGMDILLKLDLPATR 392
Solyc01g102950.3.1 337 IYEALEGNFLDSKSLSMLNPYMPNLSSSWLFQRKKQSNVPPDFINELLSANFISMK 392
Solyc10g079480.1.1 337 VANAIVQYLGSDKDHLGNELSASVWKDLWPIERRRQREFFCFGMDILLKLDLSATR 392
Solyc06g074240.2.1 321 LAEAIVEGLGSTRMIRGSQLYHRVWNGLWPLDRRCVRECYSFGMETLLKLDLKGTR 376
DCAR_022896 331 LADAIAECLGSTRMIRGSSLYHRVWNGLWPIESKCTREFYSFGMETLLKLDLNGTR 386
DCAR_020544 337 VANAIVQYLGSKKGALGNELSAEVWKDLWPIERRRQREFFCFGMDILLKLDLPGTR 392
DCAR_019750 337 IYEAISGNLLDSDNLSLLNPYMPNLSASWLFQRRKESSVSPDFINQLLCVNFQSMQ 392
AT3G10230.1 393 RFFDAFFRLFLPELLVFGLSLFSHASNTSRLEIMTKGTVPLAKMINNLVQDRD 445
AT2G32640.1 393 RLGDPVLRPFLQDIIQFGLDWSVHFFMLGLYTLLSAYIDPLLRSLEGLPSKTR 445
Solyc04g040190.1.1 393 RFFDAFFRLFLPELIVFGLSLFSHASNTSRFEIMTKGTVPLVNMINNLLQDKE 445
Solyc01g102950.3.1 393 KLGDPVLRPFLQDVIQFGLEWFGHFIMLGYYTFLSTFLDPTIRLIESFPAKMR 445
Solyc10g079480.1.1 393 RFFDAFFRLFLPELMFFGLSLFSHASNTSRLEIMTKGTFPLVTMINNLLKDTE 445
Solyc06g074240.2.1 377 RLFDAFFRLSVKELGLLSLCLFGHGSNMTRLDIVTKCPLPLVRLIGNLAIESL 429
DCAR_022896 387 NFFDAFFRLSLKELAMLSLSLFGHASNSSKMDIVTKCPAPLVKMLGNLAVETI 439
DCAR_020544 393 RFFSAFFRLFLPELFFFGLSLFSNASNTSRIEIMAKGTVPLVNMVNNLIKDRE 445
DCAR_019750 393 RLGDPVLKPFL--------LDWFGHFTMLGYYTFLSVFIDPIISSIGTLPDKTR 438
