# Supplementary figures and images for: KIPEs3: Automatic annotation of biosynthesis pathways
Source: PLoS One. 2023 Nov 16;18(11):e0294342. doi: 10.1371/journal.pone.0294342 (PMC10653506; doi:10.1371/journal.pone.0294342)

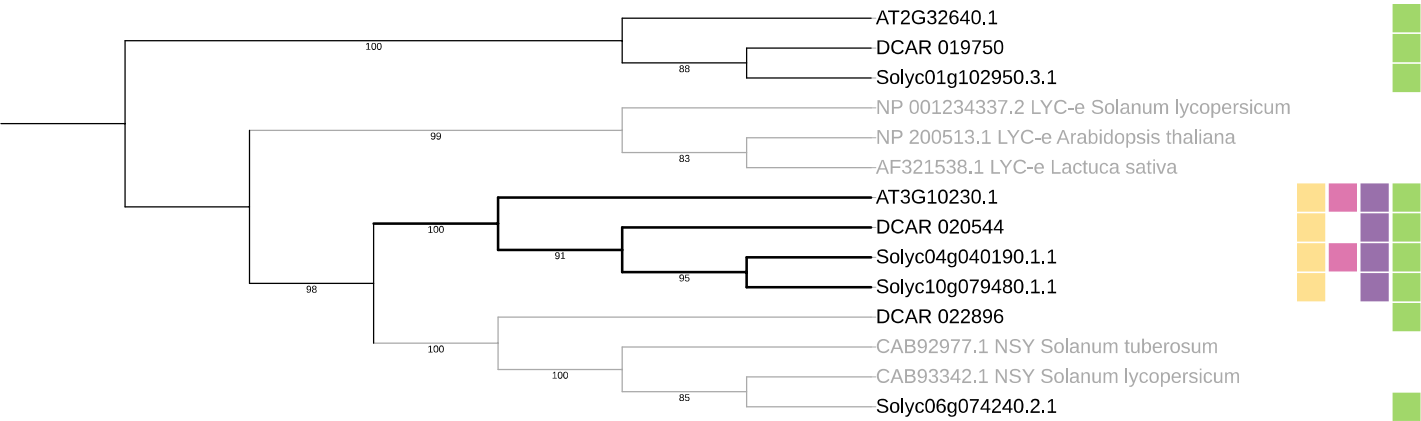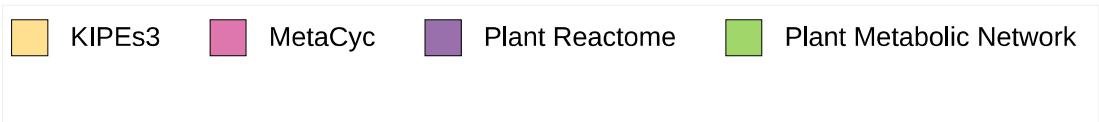

Supplement: S3 File — The highly similar sequences of Neoxanthin synthase (NSY) and Lycopene-e-cylase (LYC-e) were included as outgroup (highlighted in gray). The functional LYC-b clade was highlighted in the phylogenetic tree. (PDF) [file pone.0294342.s003.pdf]
