# Supplementary material for: KIPEs3: Automatic annotation of biosynthesis pathways
Source: PLoS One. 2023 Nov 16;18(11):e0294342. doi: 10.1371/journal.pone.0294342 (PMC10653506; doi:10.1371/journal.pone.0294342)
Supplement: S4 File — The alignment was generated using MAFFTv7. The two black boxes highlight the FLS-specific motifs “PxxxIRxxxEQP” and “SxxTxLVP.” Amino acid residues responsible for binding ferrous iron (H221, D223, and H277) and 2-oxoglutarate (R287 and S289) are marked with black and gray arrows, respectively. The residues known to be involved in the proper folding of the 2-ODD polypeptide are marked with asterisks (G68, H75, P207, and G261). The functional FLS on the basis of the presence of conserved amino acid residues are highlighted in red-yellow color. (PDF) [file pone.0294342.s004.pdf]

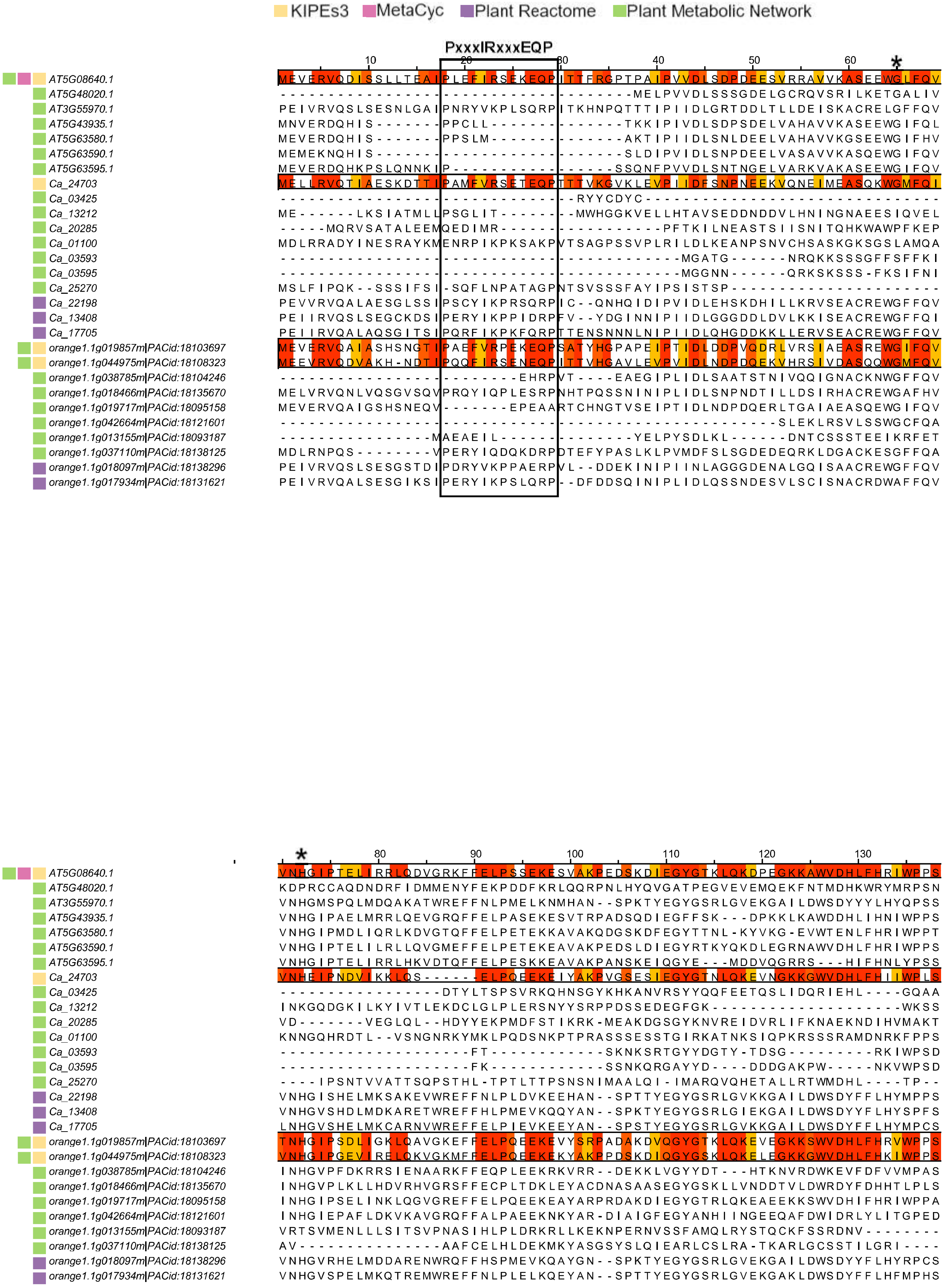
KIPEs3
MetaCyc
Plant Reactome
Plant Metabolic Network
PxxxIRxxxEQP
AT5G08640.1
AT5G48020.1
AT3G55970.1
AT5G43935.1
AT5G63580.1
AT5G63590.1
AT5G63595.1
Ca_24703
Ca_03425
Ca_13212
Ca_20285
Ca_01100
Ca_03593
Ca_03595
Ca_25270
Ca_22198
Ca_13408
Ca_17705
orange1.1g019857m|PACid:18103697
orange1.1g044975m|PACid:18108323
orange1.1g038785m|PACid:18104246
orange1.1g018466m|PACid:18135670
orange1.1g019717m|PACid:18095158
orange1.1g042664m|PACid:18121601
orange1.1g013155m|PACid:18093187
orange1.1g037110m|PACid:18138125
orange1.1g018097m|PACid:18138296
orange1.1g017934m|PACid:18131621

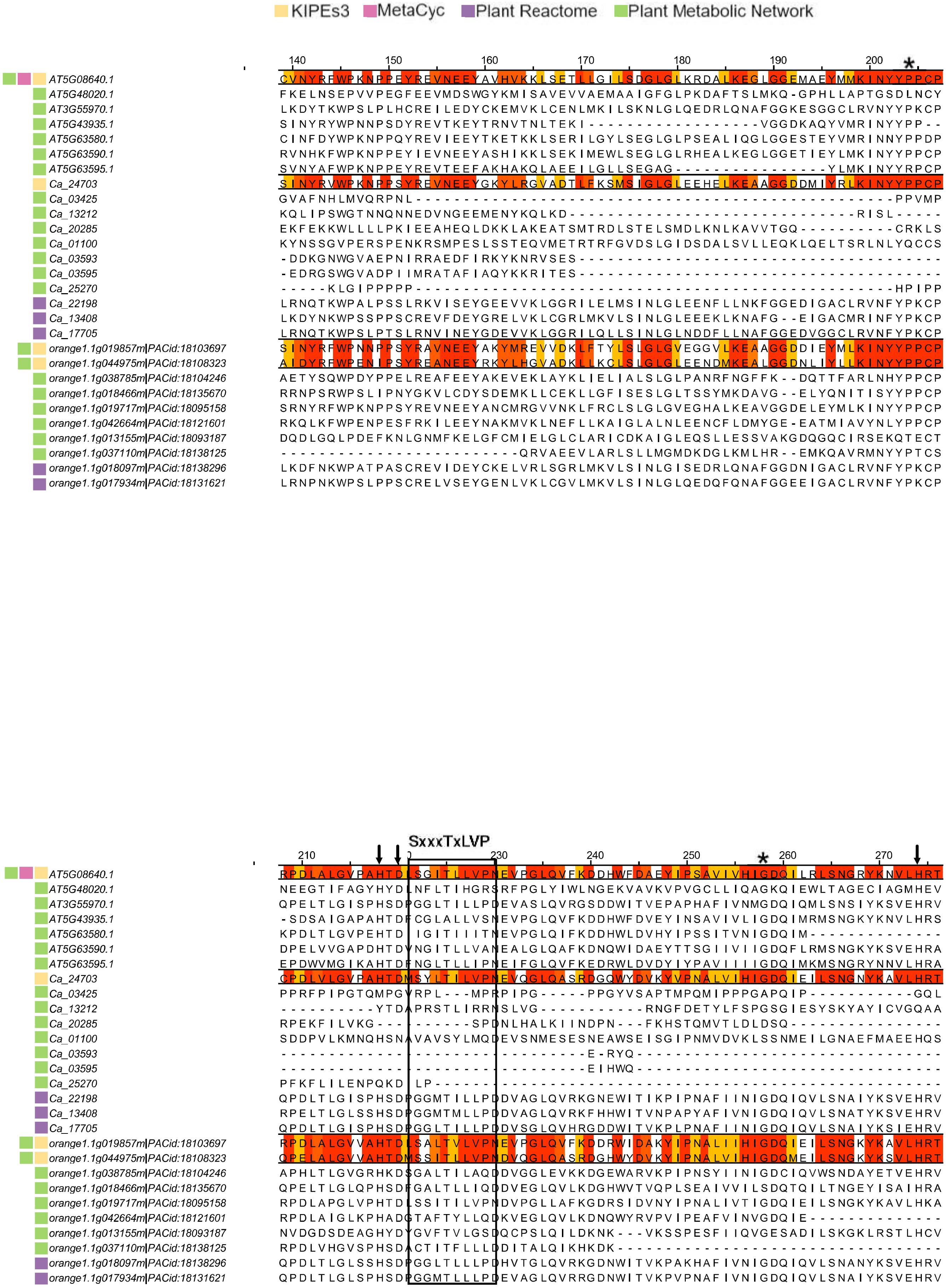

KIPEs3
MetaCyc
Plant Reactome
Plant Metabolic Network
AT5G08640.1
AT5G48020.1
AT3G55970.1
AT5G43935.1
AT5G63580.1
AT5G63590.1
AT5G63595.1
Ca_24703
Ca_03425
Ca_13212
Ca_20285
Ca_01100
Ca_03593
Ca_03595
Ca_25270
Ca_22198
Ca_13408
Ca_17705
orange1.1g019857m|PACid:18103697
orange1.1g044975m|PACid:18108323
orange1.1g038785m|PACid:18104246
orange1.1g018466m|PACid:18135670
orange1.1g019717m|PACid:18095158
orange1.1g042664m|PACid:18121601
orange1.1g013155m|PACid:18093187
orange1.1g037110m|PACid:18138125
orange1.1g018097m|PACid:18138296
orange1.1g017934m|PACid:18131621
SxxxTxLVP

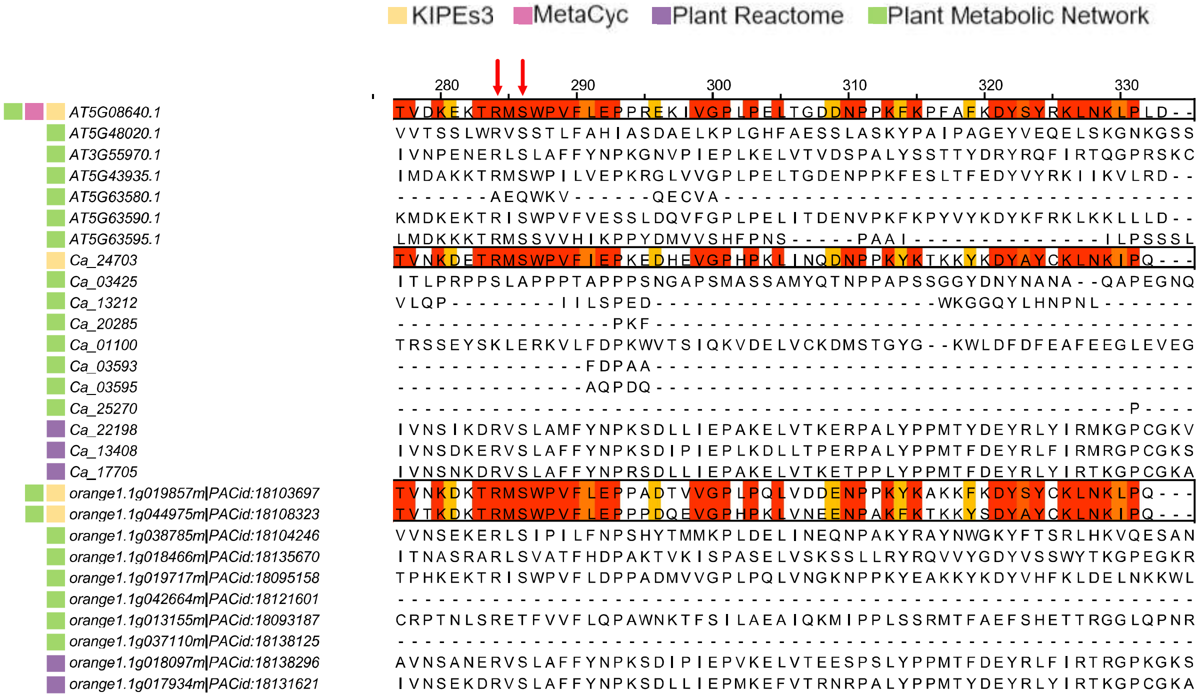
KIPEs3
MetaCyc
Plant Reactome
Plant Metabolic Network
280
290
300
310
320
330
AT5G08640.1 TVDKEKTRMSWPVFLEPPREKIVGPLPELTGDDNPPKFKPFAFKDYSYRKLNKLPLD--
AT5G48020.1 VVTSSLWRVSSTLFAHIASDAELKPLGHFAESSLASKYPAIPAGEYVEQELSKGNKGSS
AT3G55970.1 IVNPENERLSLAFFYNPKGNVPIEPLKELVTVDSPALYSSTTYDRYRQFIRTQGPRSKC
AT5G43935.1 IMDAKKTRMSWPILVEPKRGLVVGPLPELTGDENPPKFESLTFEDYVYRKIIKVLRD--
AT5G63580.1 -------AEQWKV------QECVA-----------------------------------
AT5G63590.1 KMDKEKTRISWPVFVESSLDQVFGPLPELITDENVPKFKPYVYKDYKFRKLKKLLLD--
AT5G63595.1 LMDKKKTRMSSVVHIKPPYDMVVSHFPNS-----PAAI--------------ILPSSSL
Ca_24703 TVNKDETRMSWPVFIEPKEDHEVGPHPKLINQDNPPKYKTKKYKDYAYCKLNKIPQ---
Ca_03425 ITLPRPPSLAPPPTAPPPSNGAPSMASSAMYQTNPPAPSSGGYDNYNANA--QAPEGNQ
Ca_13212 VLQP--------IILSPED--------------------WKGGQYLHNPNL-------
Ca_20285 ----------------PKF--
Ca_01100 TRSSEYSKLERKVLFDPKWVTSIQKVDELVCKDMSTGYG--KWLDFDFEAFEEGLEVEG
Ca_03593 -------------FDPAA---
Ca_03595 -------------AQPDQ---
Ca_25270 --P----
Ca_22198 IVNSIKDRVSLAMFYNPKSDLLIEPAKELVTKERPALYPPMTYDEYRLYIRMKGPCGKV
Ca_13408 IVNSDKERVSLAFFYNPKSDIPIEPLKDLLTPERPALYPPMTFDEYRLFIRMRGPCGKS
Ca_17705 IVNSNKDRVSLAFFYNPRSDLLIEPAKELVTKETPPLYPPMTFDEYRLYIRTKGPCGKA
orange1.1g019857m|PACid:18103697 TVNKDKTRMSWPVFLEPPADTVVGPLPQLVDDENPPKYKAKKFKDYSYCKLNKLPQ---
orange1.1g044975m|PACid:18108323 TVTKDKTRMSWPVFLEPPPDQEVGPHPKLVNEENPAKFKTKKYSDYAYCKLNKIPQ---
orange1.1g038785m|PACid:18104246 VVNSEKERLSIPILFNPSHYTMMKPLDELINEQNPAKYRAYNWGKYFTSRLHKVQESAN
orange1.1g018466m|PACid:18135670 ITNASRARLSVATFHDPAKTVKISPASELVSKSSLLRYRQVVYGDYVSSWYTKGPEGKR
orange1.1g019717m|PACid:18095158 TPHKEKTRISWPVFLDPPADMVVGPLPQLVNGKNPPKYEAKKYKDYVHFKLDELNKKWL
orange1.1g042664m|PACid:18121601 ---
orange1.1g013155m|PACid:18093187 CRPTNLSRETFVVFLQPAWNKTFSILAEAIQKMIPPLSSRMTFAEFSHETTRGGLQPNR
orange1.1g037110m|PACid:18138125 ---
orange1.1g018097m|PACid:18138296 AVNSANERVSLAFFYNPKSDIPIEPVKELVTEESPSLYPPMTFDEYRLFIRTRGPKGKS
orange1.1g017934m|PACid:18131621 IVNSEKDRVSLAFFYNPKSDLLIEPMKEFVTRNRPALYPPMTYDEYRLYIRTKGPCGKA
